# Clinical comparison of four types of skin incisions for skin-sparing mastectomy and immediate breast reconstruction

**DOI:** 10.1007/s00595-013-0722-2

**Published:** 2013-09-17

**Authors:** Satoki Kinoshita, Shigeya Kyoda, Akio Hirano, Tadashi Akiba, Kimihiro Nojima, Ken Uchida, Hiroshi Takeyama, Toshiaki Morikawa

**Affiliations:** 1 Department of Surgery, Jikei University Kashiwa Hospital, 163-1 Kashiwashita, Kashiwa, Chiba 277-8567 Japan; 2 Department of Plastic Surgery, Jikei University Kashiwa Hospital, 163-1 Kashiwashita, Kashiwa, Chiba 277-8567 Japan; 3 Department of Breast & Endocrine Surgery, Jikei University School of Medicine, 3-25-8 Nishi-shinbashi, Minato-ku, Tokyo, 105-8461 Japan

**Keywords:** Breast cancer, Skin-sparing mastectomy, Immediate breast reconstruction

## Abstract

**Purpose:**

Skin-sparing mastectomy (SSM) and immediate breast reconstruction (IBR) has become popular as an effective procedure for patients with early breast cancer. We herein report an overview of the four types of skin incisions used for SSM.

**Methods:**

The records of 111 consecutive breast cancer patients, who received SSM and IBR from 2003 to 2012, were reviewed retrospectively. Four types of skin incisions were used. Type A was the so-called tennis racquet incision, type B was a periareolar incision and mid-axillary incision, type C was the so-called areola-sparing with mid-axillary incision and type D was a small transverse elliptical incision and transverse axillary incision.

**Results:**

Twenty-six type A, 59 type B, 20 type C and six type D incisions were made. The average blood loss and average length of the operation during SSM were not significantly different between the four approaches. The average areolar diameter was 35 mm for type A, B and D incisions, and 45 mm for type C. There was a need for postoperative nipple–areolar complex plasty (NAC-P) in 75 % of the cases following type A, B and D incisions, and 35 % of the cases treated using type C incisions.

**Conclusion:**

The type C incision is superior with regard to the cost and cosmetic outcomes, because fewer of these patients request postoperative NAC-P.

## Introduction

The establishment of modern radical surgery for breast cancer started with standard radical mastectomy, which was first conducted by William Stewart Halsted in 1882. Since then, the surgical procedures used for breast cancer have been greatly changed from the standard radical mastectomy to breast-conserving surgery [1–12]. Today, the local control of breast cancer is the major objective of surgical treatment and is considered to be a part of systemic therapy [13], and breast-conserving surgery is the mainstay of treatment. However, about one-third of females with breast cancer still undergo mastectomy, based on the size or site of the lesion and the presence of an extensive intraductal lesion [14].

Skin-sparing mastectomy (SSM) with immediate breast reconstruction (IBR) was first reported by Toth and Lappert [15] in 1991 and is generally acknowledged to be a method that can achieve both a radical cure and excellent cosmetic outcomes. Recently, nipple-sparing mastectomy was introduced, which combines SSM with preservation of the nipple–areolar complex. However, the procedure is still controversial, and there is a lack of general consensus for breast cancer patients, although it is generally considered to be indicated as a type of prophylactic mastectomy for hereditary breast cancer.

At our hospital, we have adopted this method in cooperation with plastic surgeons and have produced excellent results since July 2003. At present, we use four types of skin incisions, depending on the individual patient situation, after we had experienced some trial and error during the initial learning period.

In the following sections, we provide an overview of the four types of skin incisions used for SSM performed by a single surgeon.

## Patients and methods

The subjects were 111 female Japanese patients who underwent SSM and IBR by a single surgeon (SK) at Jikei University Kashiwa Hospital during the period from July 2003 to December 2012.

**Fig. 1:**
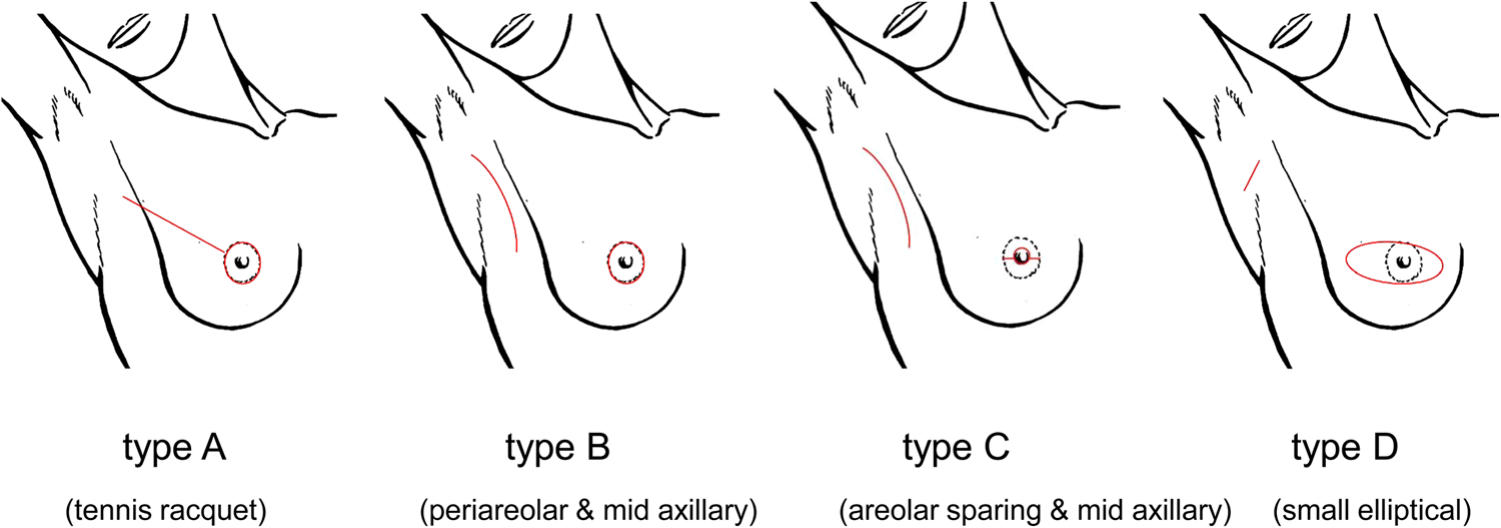
Four types of skin approach for SSM

During the SSM, removal of the nipple with/without the areola complex, biopsy scars (excluding the core needle biopsy scar) and the entire breast parenchyma was planned [16]. Immediate breast reconstruction was performed by a plastic surgeon in all patients. The patients were assigned to undergo four types of skin incisions. Type A was a periareolar incision with a lateral extension (the so-called “tennis racquet” incision), type B was a periareolar incision and axillary incision, type C included straight incisions, a small elliptical incision (base line of nipple) within areola complex (so-called “areolar sparing”) and an axillary incision and type D was a small transverse elliptical incision that contained the entire nipple–areolar complex and a transverse axillary incision (Fig. 1). When choosing the type C incision, the surgeon has to make a decision based his own intuition regarding the relationship between the breast and areola size.

The Chi square test and *t* test were used for the statistical analysis of the outcomes between the groups (*p* < 0.05).

## Results

Table 1 shows the patient demographics (26 cases were treated using type A incisions, 59 cases using type B, 20 cases using type C and six cases were treated using type D incisions) and the tumor staging determined based on the American Joint Committee on Cancer Staging System. The mean age of the patients was 44.5 years in the type A group, 47.4 years in the type B group, 50.4 years in the type C group and 43.0 years in the type D group. Early breast cancer, such as stage 0 and stage I, accounted for 54 % of the cases.

**Table 1:** Types of approach and patients, tumor characteristics

	Type A	Type B	Type C	Type D
Number of cases	26	59	20	6
Age (years)	44.5 ± 8.6 (32–62)	47.4 ± 10.1 (29–71)	50.4 ± 8.7 (39–71)	43.0 ± 5.5 (37–50)
Stage (%)				
0 (Tis)	4 (15.4)	11 (18.6)	4 (20.0)	4 (66.7)
I	9 (34.6)	20 (33.9)	8 (40.0)	0
II a	10 (38.5)	18 (30.6)	6 (30.0)	0
II b	3 (11.5)	10 (16.9)	2 (10.0)	2 (33.3)

**Fig. 2:**
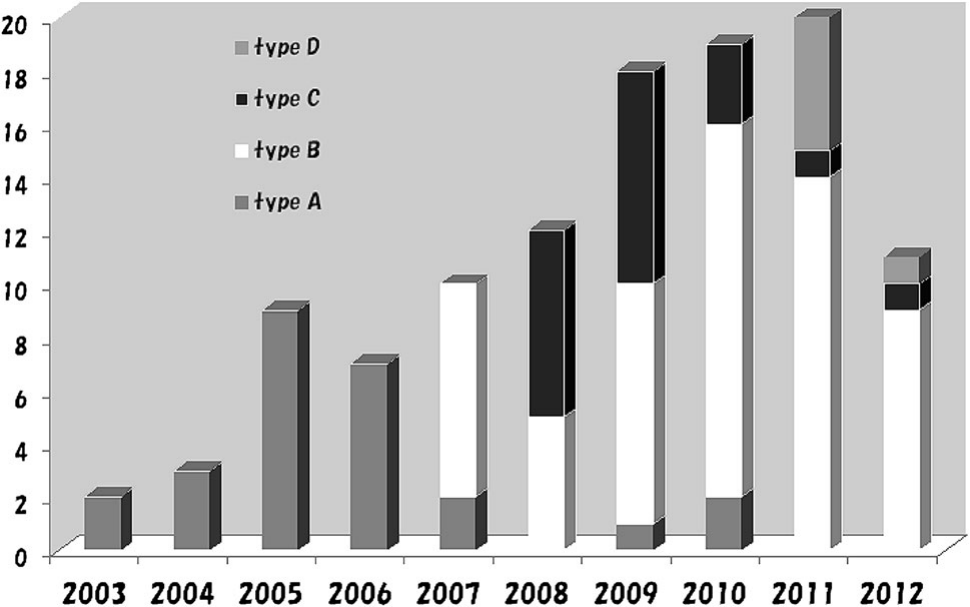
Chronological change of number and type of approach for SSM

Figure 2 shows the chronological transition of the four types of skin incisions from 2003 to 2012. While all the cases were treated using type A incisions during the first 4 years, the number of cases treated using type B and C incisions has been increasing since 2007 because they can provide better cosmetic outcomes.

Table 2 shows the average diameter of the areola, an overview of the surgical procedures and the type of reconstruction performed in the patients treated using the four types of skin incisions.

**Table 2:** Size of areola and surgical treatment

	Type A	Type B	Type C	Type D
Diameter of areola (mm)	34.0 ± 6.8 (20–50)	36.3 ± 6.8 (25–50)	44.6 ± 7.9 (35–65)	32.3 ± 4.4 (24–37)
ABD vs C			*p* < 0.000	
Reconstruction procedure (%)				
LDMC	6 (23.1)	27 (45.7)	1 (5.0)	0
TRAM	15 (57.7)	13 (22.1)	7 (35.0)	0
DIEP	5 (19.2)	19 (32.2)	11 (55.0)	0
Expander (→ implant)	0	0	1 (5.0)	6 (100.0)
Axillary management (%)				
SLNB	2 (7.7)	32 (54.2)	14 (70.0)	5 (83.3)
SLNB → ALND	1 (3.8)	13 (22.1)	4 (20.0)	1 (16.7)
ALND	23 (88.5)	14 (23.7)	2 (10.0)	0
Time for SSM (min)	138.6 ± 32.0	132.9 ± 31.4	130.0 ± 23.7	98.3 ± 28.7
Blood loss during SSM (g)	213.2 ± 110.2	188.2 ± 138.2	248.7 ± 113.7	198.3 ± 121.9

*LDMC* latissimus dorsi musuculocutaneous, *TRAM* transverse rectus abdominis musculocutaneous, *DIEP* deep inferior epigastric perforator, *SLNB* sentinel lymph node biopsy, *ALND* axillary lymph node dissection

**Fig. 3:**
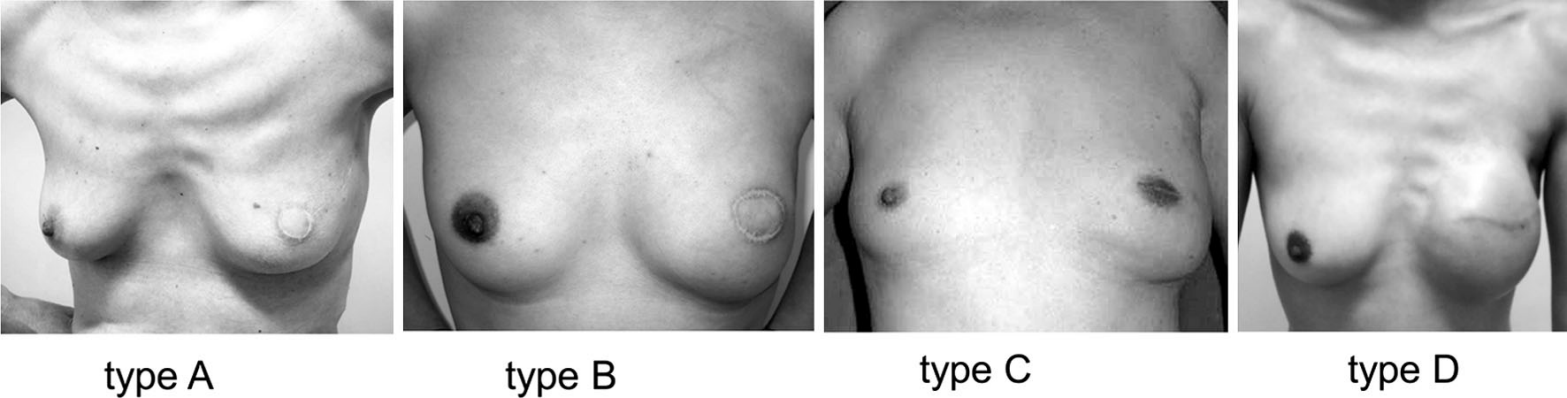
Post operative appearances of each approach for SSM and IBR

Figure 3 shows the appearance of the breast for patients treated with each type of incision after reconstruction. In this series, the most appropriate type of breast reconstruction was carried out for all patients based on their choice after adequate informed consent was obtained from them by a plastic surgeon.

The average diameter of the areola was 34.0 mm in type A cases, 36.3 mm in type B, 44.6 mm in type C and 32.3 mm in type D cases. The areola was significantly larger in type C than in the other three types (*p* < 0.000).

In the type A group, 7.7 % of the patients underwent sentinel lymph node biopsy (SLNB) alone, 3.8 % additionally underwent axillary lymph node dissection (ALND) after SLNB and 88.5 % underwent ALND. In type B cases, the percentages were 54.2, 22.1 and 23.7 %, in type C, the percentages were 70, 20 and 10 % and in type D, the percentages were 83.3, 16.7 and 0 %, respectively.

The average time required for SSM was 138.6 min in the type A group, 132.9 min for type B, 130.0 min for type C and 98.3 min in the type D group. The intraoperative blood loss was 213.2 g in the type A group, 188.2 g in type B, 248.7 g in type C and 198.3 g in the type D group.

Table 3 shows the relationship between the need for subsequent nipple–areolar complex plasty (NAC-P) and the type of incision. Postoperative NAC-P was requested in 19 cases (73.1 %) in the type A group, 44 cases (74.6 %) in the type B group, seven cases (35 %) in the type C group and five cases (83.3 %) in the type D group. The number of patients requesting subsequent NAC-P was significantly lower in the type C group than that in the other three groups (*p* = 0.001).

**Table 3:** Types of approach and nipple areolar complex plasty

	Type A	Type B	Type C	Type D
NAC-plasty (%)				
Desired	19 (73.1)	44 (74.6)	7 (35.0)	5 (83.3)
Not desired	7 (26.9)	15 (25.4)	13 (65.0)	1 (16.7)
ABD vs C			*p* = 0.001	

*NAC* nipple areolar complex

## Discussion

SSM with IBR has rapidly spread during the past 20 years, and its origin dates back to subcutaneous mastectomy, which was first performed by Freeman in 1962 [17].

During SSM, the nipple–areolar complex and all biopsy scars, excluding the core needle biopsy scar, are resected; the inframammary fold and most of the native breast skin are preserved, and the entire breast parenchyma is removed. SSM is usually followed by IBR, which provides better cosmetic outcomes, and the anesthetic risk and the patient’s emotional trauma from the loss of a breast are reduced, which ultimately also leads to a better cost effectiveness of the treatment [18, 19].

In view of the anatomical course of the ducts, resection of the nipple–areolar complex has been considered to be essential, because the tumor cells may spread to the adjacent ducts. The involvement of tumor cells at the nipple–areolar complex is reported to occur in about 3–10 % of cases, except for the extremely high percentage of 58 % reported in one study [14, 16]. On the other hand, Simmons et al. [20] examined the nipple and areola separately and reported that areolar involvement was seen in just 0.9 % of cases. At our institution, we have been trying an approach that uses the type C skin incision since 2008, while taking into account the information obtained from preoperative contrast-enhanced CT/MRI to achieve better cosmetic outcomes, and obtained similar positive outcomes as were seen in the study by Simmons et al. [21], although our study period was relatively short [22].

The average areolar diameter of patient who underwent type C incisions (44.6 mm) was significantly larger than that of the patients who underwent type A, B and D incisions (32.3–36.3 mm), and the smaller diameter may make it difficult to ensure a clear operating field for these three types of incisions. Based on the average breast size in Japanese females, we considered that areolar-sparing mastectomy could be performed safely in patients with a long axis of the areola measuring at least 4 cm. In addition, type C is considered to be far superior with regard to the cost and cosmetic outcomes because the number of patients who wanted to undergo postoperative nipple–areolar plasty after a type C incision (35 %) was significantly lower than that of patients who were treated using type A, B and D incisions (73.1–83.3 %).

In most of the cases treated with type C incisions, the defects after the removal of the nipple were small (5–10 mm in diameter), and are relatively unremarkable, so the surgical scars within the areola are not noticeable (Fig. 3). Therefore, especially in the cases with small nipples, patients do not desire further operations, such as NAC-P. We usually have performed NAC-P after an interval of 6 months or longer, because there are sometimes minor changes in the nipple symmetry and also because the blood supply to the flap and its viability has to be confirmed.

**Fig. 4:**
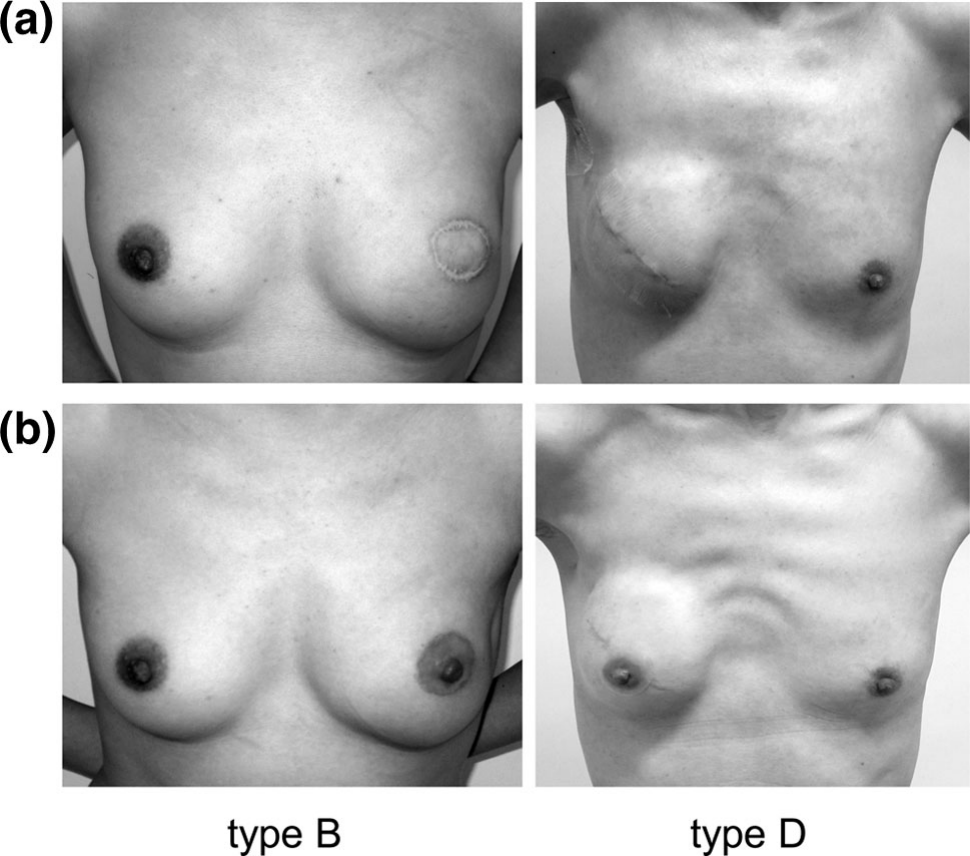
Post operative appearance of type B and D without applying nipple areolar complex made with silicon material (**a**), and with applying nipple areolar complex made with silicon material (**b**)

At our hospital, the total cost for NAC-P is approximately ¥660,000, including the surgeon’s fee, and requires hospitalization of the patient for about 10 days and a tattoo on the areola (Table 4). According to the medical insurance system in Japan, the individual payment for the upper limit of 30 % is calculated to be around ¥200,000. Additionally, after spring 2009, a nipple–areolar complex made of silicone has been tested and is considered effective especially for type A, B and D incisions (Fig. 4). Performing breast reconstruction with implants has been difficult in Japan, because the medical insurance system in Japan does not cover such procedures. This had led to 94 % of the patients undergoing breast reconstruction with autogenous tissue. However, beginning in the spring of 2013, implants have been covered by the national insurance program, and an expansion of the choices of breast reconstruction is expected.

**Table 4:** Cost for nipple areolar complex plasty in our hospital

NAC plasty	Cost for operation fee	¥73,500
	Cost for ten days admission	¥526,500
	Cost for tatoo	¥80,000
	Total	¥660,000
NAC made with silicon material		¥80,000

*NAC* nipple areolar complex

In this investigation, since many patients who had undergone the type A approach were treated between 2003 and 2006, more patients with ALND were found. At present, since the majority of the breast-cancer patients are in an early stage, SLNB is indicated for most patients.

Although no prospective randomized study about a consensus that compares SSM and non-skin-sparing mastectomy (NSSM) has been conducted so far, it is commonly acknowledged that the local control, prognosis and risk of complications are similar for SSM and NSSM, at least for patients with stage II or earlier breast cancer [22]. SSM is still considered to be contraindicated for inflammatory breast cancer and breast cancer with skin invasion. Although there have been some studies on the usefulness of SSM in locally advanced breast cancer [23, 24], its application is still controversial. Nonetheless, SSM is considered to be a surgical procedure that can be of great benefit to patients with relatively early-stage breast cancer who are potential candidates for breast conservation, but who are not suitable for breast-conserving surgery. We will continue our efforts to ameliorate the cosmetic results and curability of breast cancer.

## Conclusion

We herein compared and investigated the four types of approaches used in the patients treated with SSM and IBR.

Many of the patients who underwent the type C approach did not need NAC-P; therefore, type C approach is considered to be more effective, not only in terms of the cosmetic results, but also in terms of cost-effectiveness.

## References

[1] Halsted WS (1907). The results of radical operations for the cure of carcinoma of the breast. Ann Surg.

[2] Haagensen CD (1986). Disease of the breast.

[3] Fisher B, Fisher ER (1967). Barrier function of lymph nodes to tumor cells and erythrocytes. Cancer.

[4] Fisher B, Fisher ER (1966). Transmigration of lymph nodes by tumor cells. Science.

[5] Fisher B, Redmond C, Fisher ER (1980). The contribution of recent NSABP clinical trials of primary breast cancer therapy to an understanding tumor biology—an overview of findings. Cancer.

[6] Veronesi U, Valagussa P (1981). Inefficacy of internal mammary nodes dissection in breast cancer. Cancer.

[7] Wilson RE, Donegan WL, Mettlin C, Natarajan N, Smart CR, Murphy GP (1984). The 1982 national survey of carcinoma of the breast in the United States by the American College of Surgeons. Surg Gynecol Obstet.

[8] Sono H, Fukuda M (2005). Results of questionnaires concerning breast cancer surgery in Japan 1980–2003. Breast Cancer.

[9] Fisher B, Bauer M, Margolese R, Poisson R, Pilch Y, Redmond C (1985). Five-year results of randomized clinical trial comparing total mastectomy and segmental mastectomy with or without radiation in the treatment of breast cancer. N Engl J Med.

[10] Veronesi U, Cascinelli N, Mariani L, Greco M, Saccozzi R, Luini A (2002). Twenty-year follow-up of randomized study comparing breast-conserving surgery with radical mastectomy for early breast cancer. N Engl J Med.

[11] Fisher B, Anderson S, Bryant J, Margolese RG, Deutsch M, Fisher ER (2002). Twenty-year follow-up of a randomized trial comparing total mastectomy, lumpectomy, and lumpectomy plus irradiation for the treatment of invasive breast cancer. N Engl J Med.

[12] Kijima Y, Yoshinaka H, Hirata M, Mizoguchi T, Ishigami S, Arima H (2013). Immediate reconstruction using a modified inframammary adipofascial flap after partial mastectomy. Surg Today.

[13] Fisher B (1999). From Halsted to prevention and beyond: advances in the management of breast cancer during the twentieth century. Eur J Cancer.

[14] Patani N, Devalia H, Anderson A (2008). Oncological safety and patient satisfaction with skin-sparing mastectomy and immediate reconstruction. Surg Oncol.

[15] Toth BA, Lappert P (1991). Modified skin incision for mastectomy: the need for plastic surgical input in preoperative planning. Plast Reconstr Surg.

[16] Simmons RM, Adamovich TL (2003). Skin sparing mastectomy. Surg Clin N Am.

[17] Rainsbury RM (2006). Skin-sparing mastectomy. Br J Surg.

[18] Singletary SE, Kroll SS (1997). Skin-sparing mastectomy with immediate breast reconstruction. Adv Surg.

[19] Lanitis S, Tekkis PP, Sgourakis G, Dimopoulos N, Al Mufti R, Hadjiminas DJ (2010). Comparison of skin-sparing mastectomy versus non-skin-sparing mastectomy for breast cancer. A meta-analysis of observational studies. Ann Surg.

[20] Simmons RM, Brennan RN, Christos P, King V, Osborne M (2002). Analysis of nipple/areolar involvement with mastectomy: can the areola be preserved?. Ann Surg Oncol.

[21] Simmons RM, Hollenbeck ST, LaTrenta GS (2004). Two-year follow-up of areolar sparing mastectomy with immediate reconstruction. Am J Surg.

[22] Kinoshita S, Nojima K, Takeishi M, Imawari Y, Kyoda S, Hirano A (2011). Retrospective comparison of non-skin-sparing mastectomy and skin-sparing mastectomy with immediate breast reconstruction. Int J Surg Oncol.

[23] Foster RD, Esserman LJ, Anthony JP, Hwang ES, Do H (2002). Skin-sparing mastectomy and immediate breast reconstruction: a prospective cohort study for the treatment of advanced stages of breast carcinoma. Ann Surg Oncol.

[24] Lim W, Ko BS, Kim HJ, Lee JW, Eom JS, Son BH (2010). Oncological safety of skin sparing mastectomy followed by immediate reconstruction for locally advanced breast cancer. J Surg Oncol.

